# Complex ruptures of the quadriceps tendon: a systematic review of surgical procedures and outcomes

**DOI:** 10.1186/s13018-021-02696-9

**Published:** 2021-09-04

**Authors:** Francesco Oliva, Emanuela Marsilio, Filippo Migliorini, Nicola Maffulli

**Affiliations:** 1 Department of Musculoskeletal Disorders, Faculty of Medicine and Surgery, University of Salerno, 84084 Baronissi, Italy; 2 Clinica Ortopedica, Ospedale San Giovanni di Dio e Ruggi d’Aragona, 84131 Salerno, Italy; 3 Department of Orthopaedic, Trauma, and Reconstructive Surgery, RWTH Aachen University Hospital, Pauwelsstraße 31, 52074 Aachen, Germany; 4 Centre for Sports and Exercise Medicine, Barts and The London School of Medicine and Dentistry, Mile End Hospital, 275 Bancroft Road, London, E1 4DG UK; 5 School of Pharmacy and Biotechnology, Keele University School of Medicine, Thornburrow Drive, Stoke on Trent, Keele, England

**Keywords:** Quadriceps tendon, Rupture, Rerupture, Inveterated rupture, Neglected rupture, Chronic rupture, Chronic quadriceps tendon rupture and TKA, Surgery

## Abstract

**Background:**

Chronic ruptures, ruptures following total knee arthroplasty (TKA), and re-ruptures of the quadriceps tendon (QT) are rare. A systematic review of the current literature was conducted on their treatment and outcome to provide evidence-based indications for their management.

**Methods:**

We searched published articles in English on chronic ruptures of QT, QT ruptures that occurred after TKA, and re-ruptures in PubMed, Scopus, and Google Scholar up to January 2021. Twenty-five articles were included following the Preferred Reporting Items for Systematic Reviews and Meta-Analyses (PRISMA) guidelines.

**Results:**

Data from 25 articles (97 patients) with a mean age of 57 were retrieved. Patients were classified into three groups depending on the type of rupture: 16 patients suffered chronic QTR, 78 a QTR after a TKA, and 9 patients reported a re-rupture. The most frequent surgical approaches were different for each group: Codivilla’s Y-V technique and end-to-end sutures were the most commonly used in the chronic tears group (62.5%), synthetic MESH was the most frequent choice in QTR after a TKA group (38 patients, 53%), while end-to-end sutures were the first choice in the re-rupture group (4 patients, 44%).

**Conclusions:**

Complex ruptures of the QT can be chronic ruptures, re-ruptures, or ruptures occurring after TKA. The choice of the best surgical technique depends on the macroscopic quality of the tendon stumps rather than the timing of intervention. Evidence-based preventive and therapeutic strategies should be developed.

**Supplementary Information:**

The online version contains supplementary material available at 10.1186/s13018-021-02696-9.

## Introduction

The tendinous insertion of the QT is composed of three distinct planes: the most superficial contains the rectus femoris, in the middle plane lie the vastus medialis and lateralis, and the deepest plane includes the vastus intermedius [1]. In chronic ruptures of the quadriceps tendon, this multi-layered organization is gradually lost, and retraction of the tendon collagen fibers occurs [1]. Quadriceps tendon rupture (QTR) is uncommon, with an annual incidence of 1.37 patients per 100.000 persons, affecting mainly middle aged males (M:F = 4.2:1, Mean age: 51.1 years) [2] . Most case series include patients with traumatic QTRs, and the main mechanism of injury reported is a sudden eccentric contraction of the quadriceps muscle complex, usually to prevent a fall [3]. Although a traumatic injury is often described, spontaneous, sometimes even bilateral ruptures, can occur in patients with predisposing systemic conditions such as chronic renal failure, diabetes, rheumatoid arthritis, hyperparathyroidism, and gout [4]. The use of drugs such as steroids and fluoroquinolones can lead to a QTR, and their use should be investigated in each patient [5]. QTR can also occur as a complication of a total knee arthroplasty, with extensor mechanism disruption, with an incidence between 0.1 and 3% of all TKAs [6, 7]. These ruptures can be extremely difficult to manage, with no consensus regarding the optimal technique. Primary repair is usually performed in acute QTRs after TKA, while in chronic tears after TKA augmentation, reconstruction is likely the most suitable option to restore the substance loss [8]. Different anatomical sites of the tendon can be affected. QTR can occur at the tendon-bone junction, or 1-2 cm proximal to the superior pole of the patella, a hypovascular area of the tendon [9]. Clinical history and physical examination play a crucial role in QTR diagnosis. Clinical signs are pain proximal to the patella, inability to actively extend the knee, and a palpable suprapatellar gap [10]. In suspected QTRs, radiography, ultrasound scan, and MRI have all been used to corroborate the clinical diagnosis [11]. The surgical approach for acute QTR is well established [12, 13]. Several surgical strategies have been suggested. The most commonly used and well described are transosseus sutures and anchors, but it is still unclear which technique offers the best postoperative advantages because of the limited number and quality of available studies [14]. A chronic tendon rupture has typically not been diagnosed or treated for at least 3 weeks [15]. The choice of the best surgical technique can be challenging, and it depends on the macroscopic quality of the tendon stump rather than the timing of intervention [16]. In chronic QTR, a large defect or fibrotic tendon retraction can be found, and direct repair, with transosseous sutures or anchors, is often not achievable.

Several augmentation techniques have been developed to restore the anatomy and functions of the quadriceps tendon [1, 3, 17–19]. However, there are no randomized controlled trials regarding the outcome of these procedures, and hence a lack of standard surgical protocols. A systematic review of the literature was performed to investigate the epidemiology, treatment and outcomes of chronic and QTRs after TKA, and re-ruptures of the QT.

## Methods

### Search strategy

This systematic review was conducted according to the Preferred Reporting Items for Systematic Reviews and Meta-Analyses: the PRISMA guidelines [20]. The literature search was guided by the following points:

- Problem: complex quadriceps tendon ruptures;
- Intervention: end-to-end sutures, V-Y lengthening, tendon grafts;
- Comparison: chronic ruptures vs chronic ruptures after TKA vs re-ruptures of QT;
- Outcome: PROMs and complications.

### Literature search

Two authors (**;**) independently performed the literature search up to June 2021. PubMed and Google scholar were accessed. Embase and Web of Science were also accessed to identify further articles. The following keywords were used in combination: *quadriceps tendon, rupture, re-rupture, inveterate rupture, neglected rupture, chronic rupture, delayed rupture, chronic quadriceps tendon rupture, total knee arthroplasty (TKA), surgery, clinical outcomes.* The same authors screened the titles resulting from the search in a separate fashion and accessed the full text of the articles of interest. Manual cross-reference of the bibliographies of the full-text articles was also performed. Disagreements were resolved by a third experienced researcher (**).

### Eligibility criteria

All the published clinical studies in English reporting the incidence, risk factors, treatment, and complications of complex QTRs were accessed. Level I to IV of evidence articles, according to the Oxford Centre of Evidence-Based Medicine [21], were considered. Reviews, technical notes, comments, letters, editorials, protocols, and guidelines were not eligible, nor were biomechanical, animal, and cadaveric studies. Studies reporting data on acute and intraoperative ruptures that occurred during TKA were excluded. Studies reporting QTRs following revision surgery for TKA were excluded. Only articles reporting quantitative data under the outcomes of interest were considered for inclusion.

### Outcome of interests

Data extraction was performed by two authors (**;**). The following information was extracted from each article: mechanism of injury, mean range time before surgery, type of lesion/re-rupture, associated injuries and comorbidities, type of surgery, follow-up, complications.

## Results

**Fig. 1:**
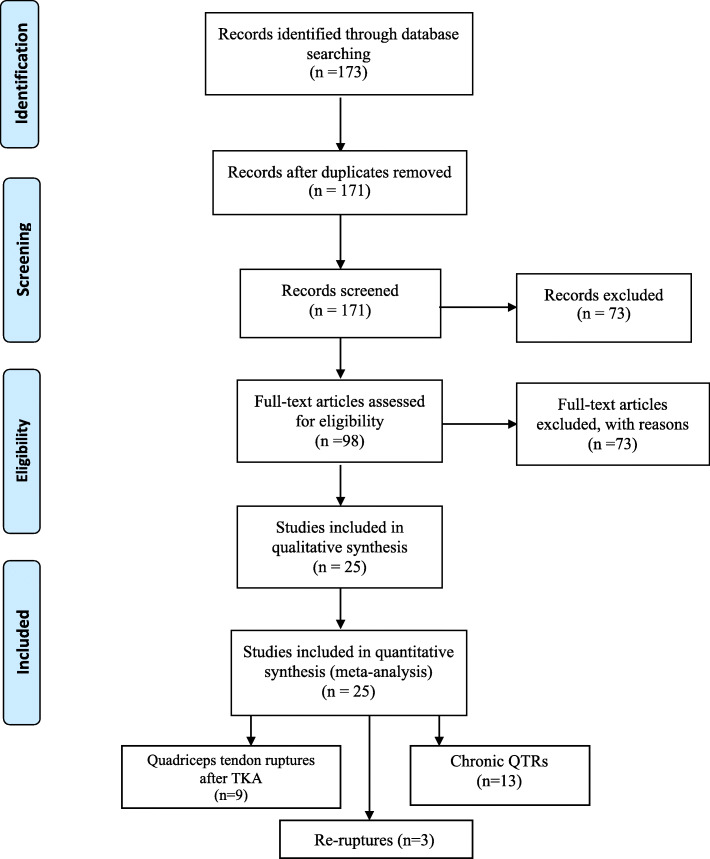
Study selection, retrieval and inclusion and exclusion reasons

**Fig. 2:**
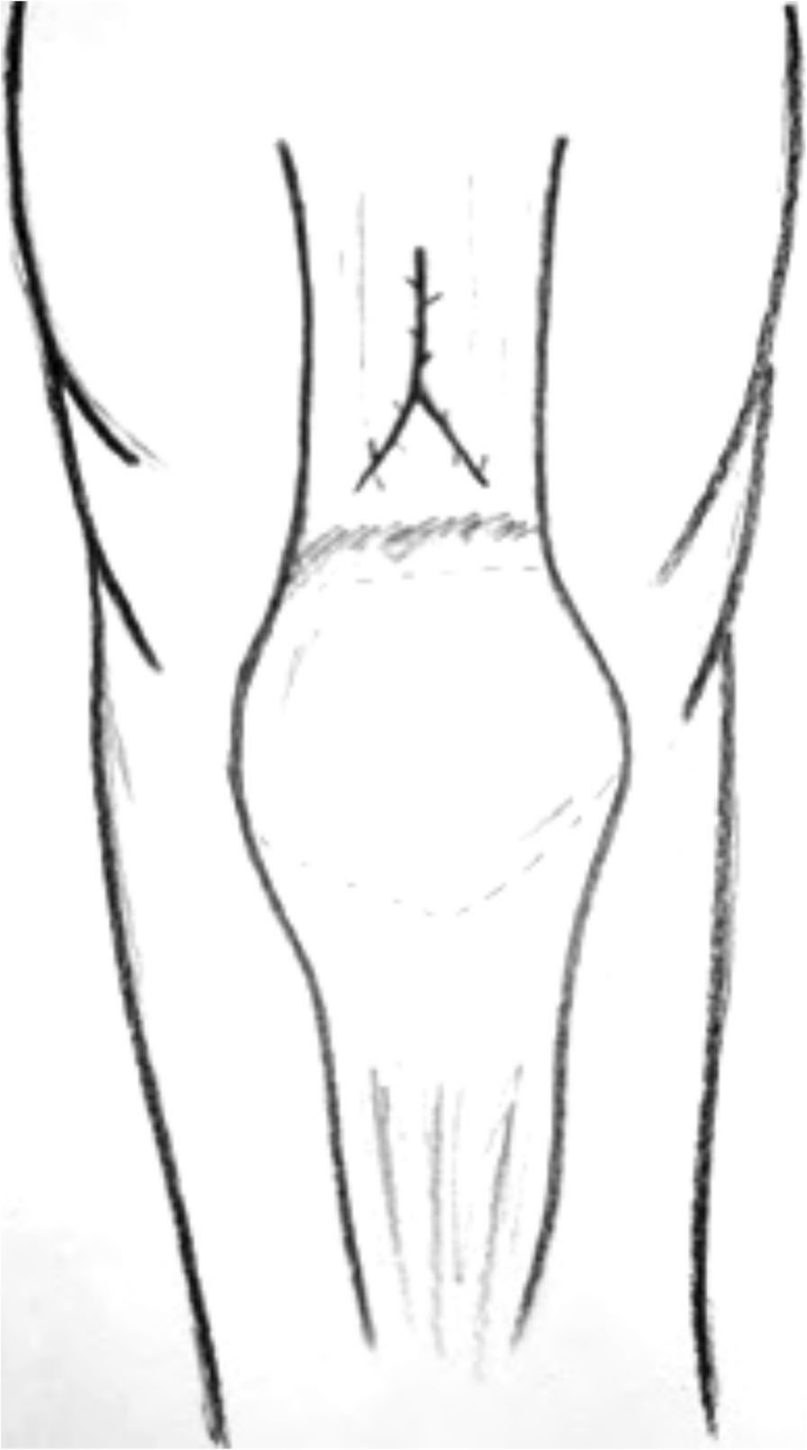
Simultaneous chronic rupture of quadriceps tendon in a patient affected by tertiary hyperparatiroidism

**Fig. 3:**
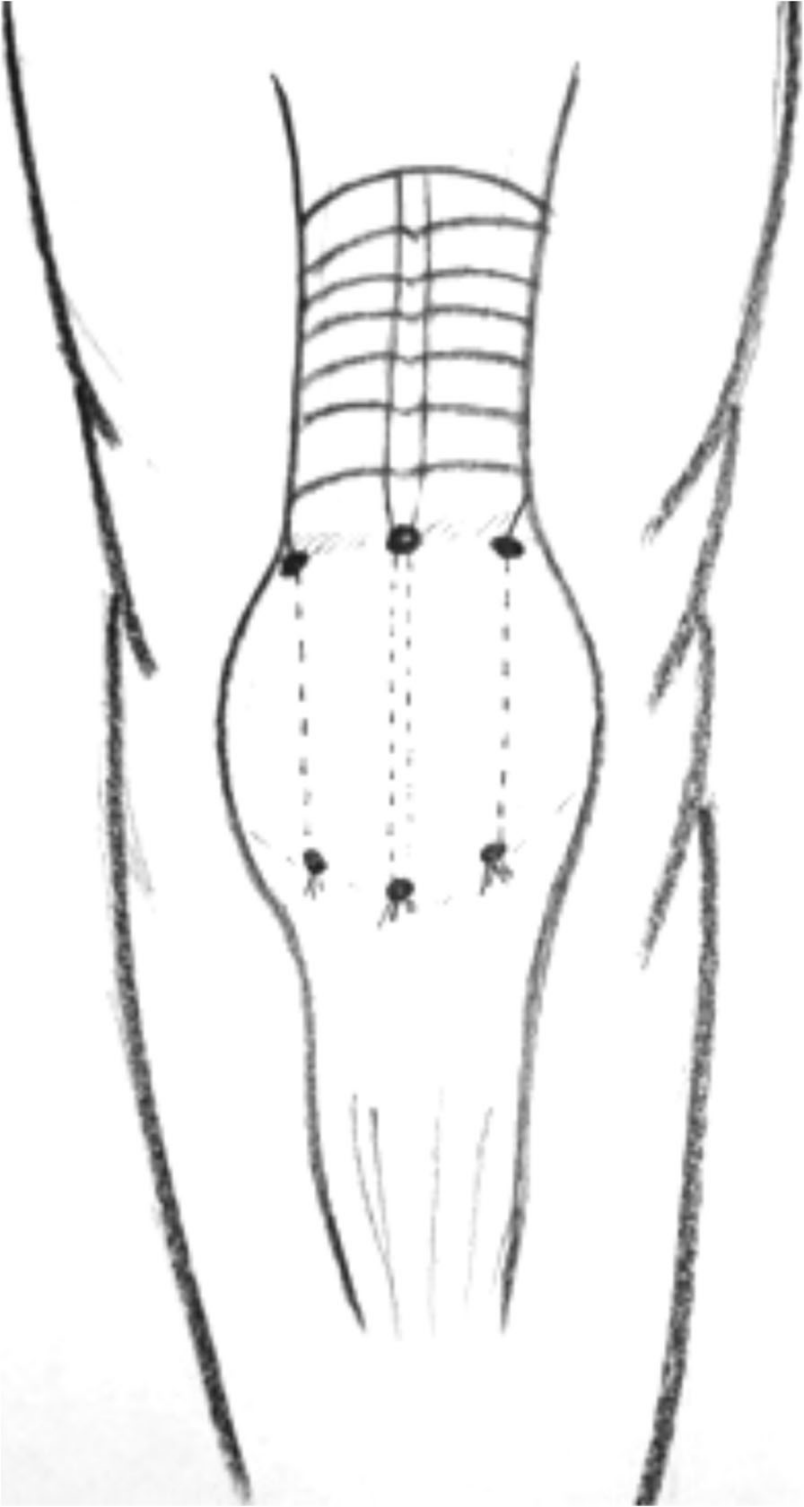
Simultaneous chronic rupture of contra-lateral patellar tendon in a patient affected by tertiary hyperparatiroidism

**Fig. 4:**
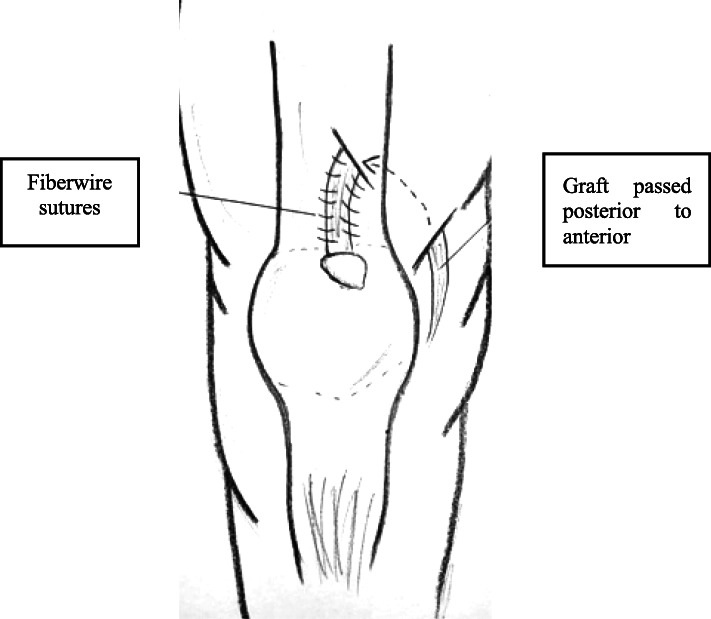
The hemisoleus rotational flap provides a novel superior autograft reconstructive option for the treatment of chronic extensor mechanism disruption

**Fig. 5:**
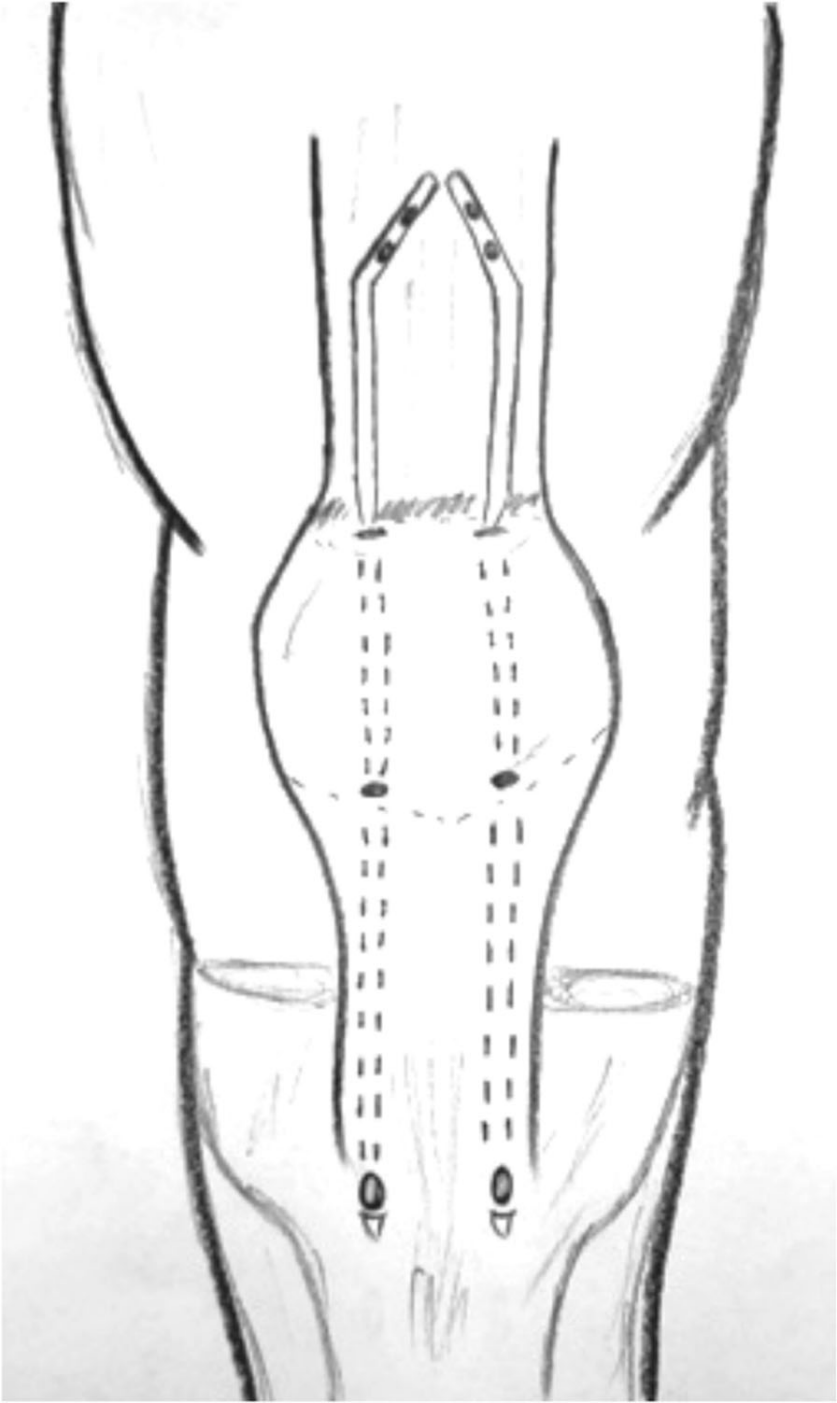
ipsilateral simultaneous ruptures of patellar and quadriceps tendon

**Fig. 6:**
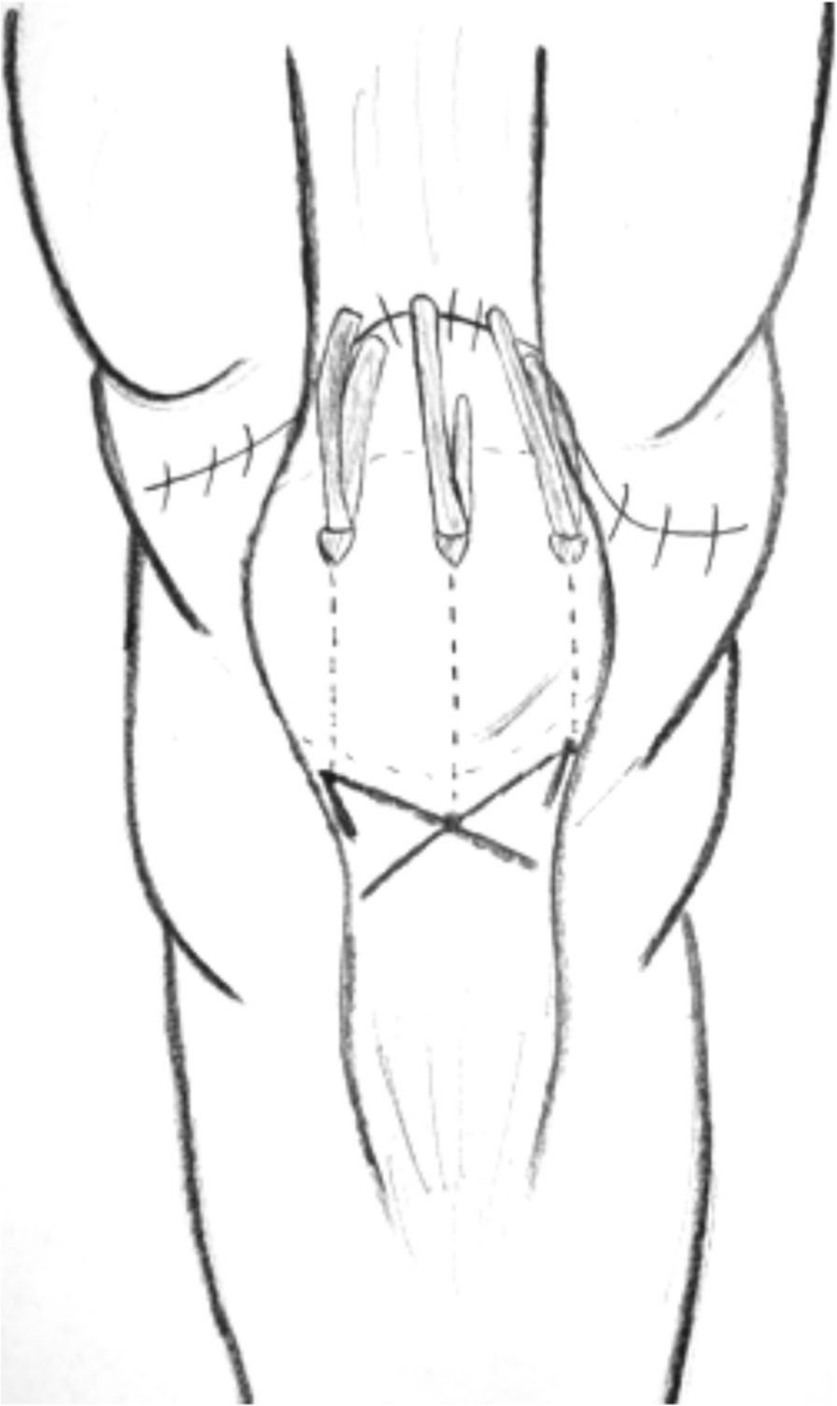
Autologous hamstring tendon used for revision of quadiceps tendon tears

**Fig. 7:**
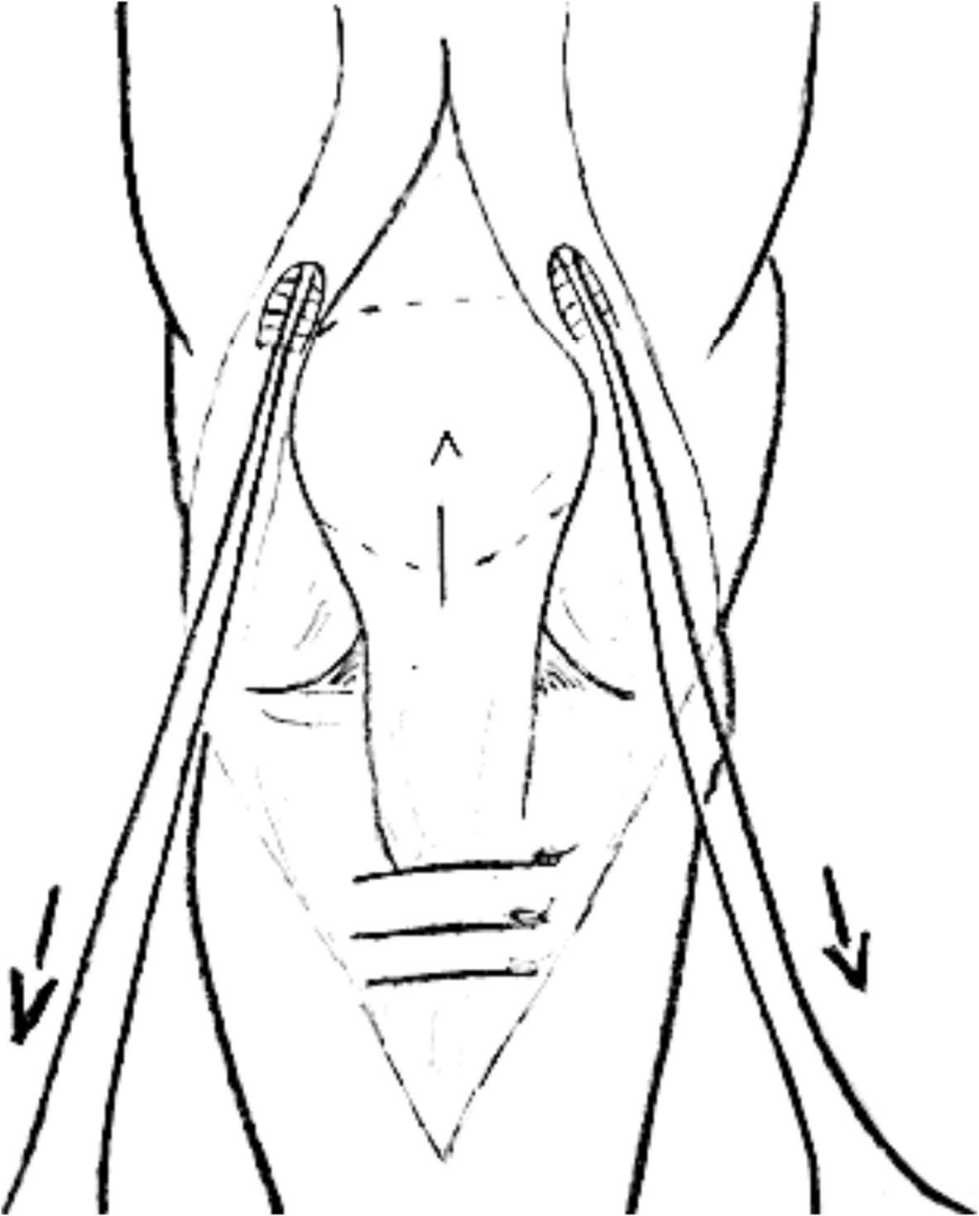
Knee osteoarthritis with chronic quadriceps tendon rupture treated with total knee arthroplasty and extensor mechanism allograft reconstruction

**Fig. 8:**
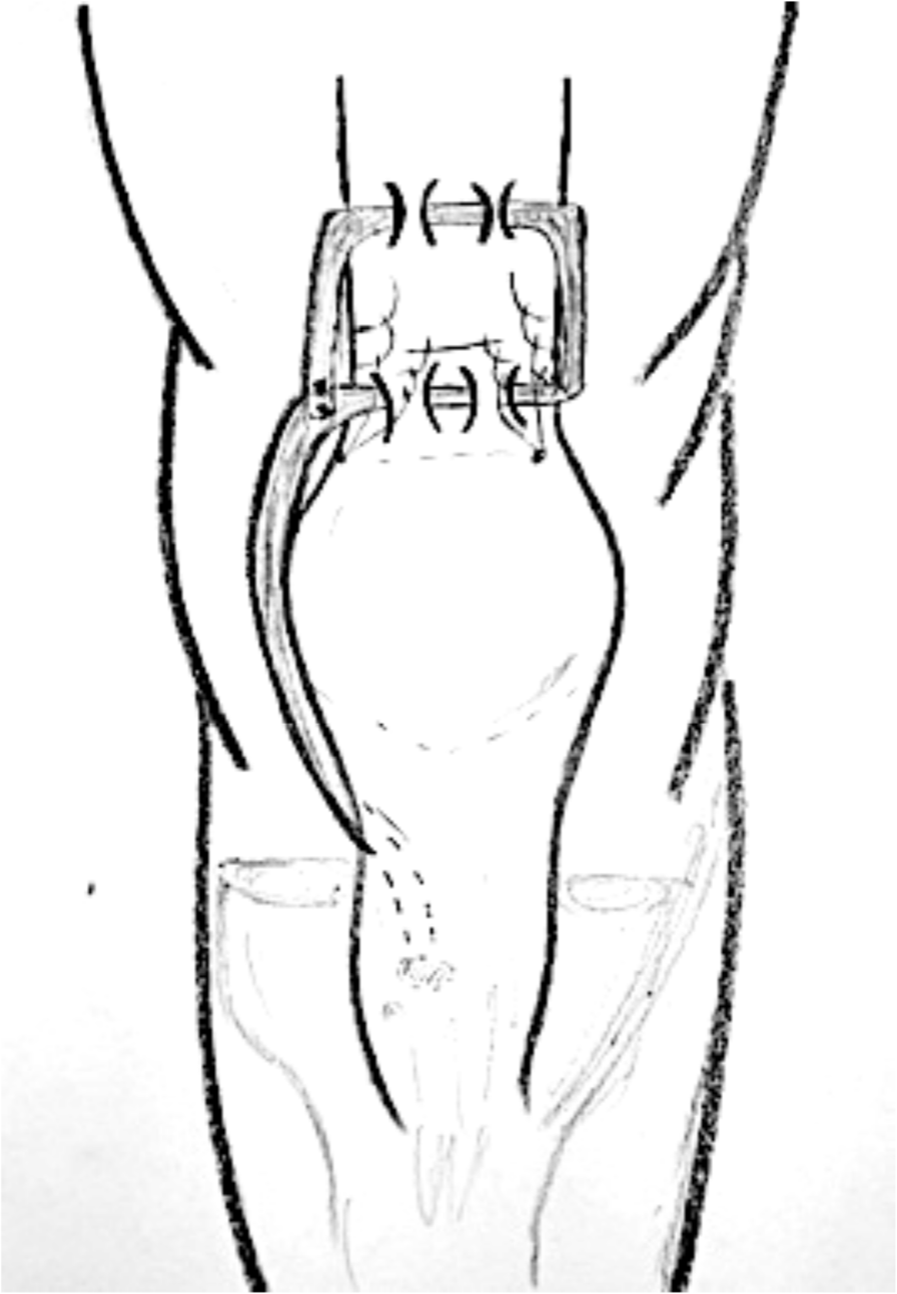
Extensor mechanism reconstruction with use of Marlex Mesh

After our initial literature search, a total of 173 potentially relevant citations were identified. Title and abstract review excluded 148 articles on the basis of irrelevant pathology or non-English language. A total of 25 articles for a total of 97 patients were eventually included in the present review. Study selection, retrieval and inclusion, and exclusion reasons are shown in the flowchart in Fig. 1. The relevant reference, the methods and the data collected from the included articles are shown in Table 1. Following the collection of data from the articles included in the present systematic review, patients were divided into three groups: chronic QTRs, QTRs that occurred after primary TKA, and re-ruptures (Figs. 2 and 3). The chronic QTR group included 16 patients (20 QTRs), and males were more represented (11 patients, 68.75%) with a mean age of 41. The female group included five patients (31.25%) and was older (mean age of 54 years). Seventy-two patients (73 QTRs) experienced a QTR after primary TKA, of whom 43 were females (60%) with a mean age of 58.7 years, while the male group was smaller (29 patients, 40%) with a mean age of 66 years. No significant difference was evidenced in relation to the surgical technique used for TKA. The re-rupture group included nine patients, with a mean time range from the first QTR of 322.5 days. Five patients (55.5%) were males with a mean age of 55.2 years, and four (44.5%) were females with a mean age of 70 years (Figs. 4, 5, 6, 7, and 8). Demographic characteristics and comorbidities of the included patients were reported in Table 2. Chronic quadriceps tendon reconstruction was performed using different surgical procedures: in the chronic QTR group, six patients were treated with grafts [three autografts (18.75%) and three allografts (18.75%)], in five patients (31.25%) tendon augmentation was performed using Codivilla’s V/Y technique, while in five patients (31.25%), end-to-end sutures were performed (Table 3). Synthetic mesh reconstruction was the most commonly used treatment in the QTR after the TKA group (38 patients, 53%); in 22 patients (30%), tendon augmentation was performed using Codivilla’s V/Y technique, three patients (4.5%) were treated with end-to-end sutures, while nine patients received allografts (12.5%). In this group, synthetic materials were chosen to restore tendon function in 38 patients (49%), and different materials were used: Marlex Mesh (27 patients, 71%), propylene mesh (seven patients, 18%), MUTARS mesh (three patients, 8%) and Keio-Leeds ligament in one case (3%). Re-ruptures were treated with end-to-end sutures in four cases (44%), with grafts in four cases [three autografts (34%) and one allograft (11%)], and with Codivilla’s V/Y technique in one patient (11%). Rehman et al. used a semitendinosus and gracilis autograft and reinforced it with a synthetic Mesh in one case (11%) of re-rupture. All the patients included in the present systematic review recovered active extension, even though at the last follow-up, some patients presented an extension lag. Sixteen patients (20.5%) from the TKA group reported an extension lag with a mean value of 6.3°, two patients (12.5%) from the chronic group presented an extensor lag with a mean value of 22.5°, and three patients (34%) who underwent a re-rupture reported a mean value of 7.3°. Forty-nine patients (50%) showed full active flexion, while 48 patients (50%) regained 90–130° flexion: 40 patients (51%) from the TKA group reported a mean 104° flexion, six patients (37.5%) from the chronic group reported a mean 103.6° flexion while two patients (22%) with re-ruptures reported a mean 90° flexion. In two patients, the findings at post-operative follow-up were not recorded. One patient with a chronic tear (6.25%) developed a skin ulcer at the level of the tibial fixation ten months after surgery. This was treated with surgical removal of the cerclage wire, with uneventful skin healing in 2 weeks [22]. As a result of QT repair after TKA, one patient (1%) developed a hematoma resulting in a delayed wound healing [23]. Furthermore, 13 patients (13.5%) reported severe complications: 9 (9%) re-ruptures of the QT, three (3%) deep infections that led in two cases to re-rupture and in one case to a graft failure, while one patient (1%) showed a chronic recurvatum. Several scoring systems were used to assess the functional outcome of the surgical technique, including the Lysholm Score, the International Knee Documentation Committee (IKDC), and the Knee Society Score. However, only few studies referred to these PROMs; thus, further statistical considerations were not possible.

**Table 1:** Characteristics of the studied included

Nr. of reference	No. of patients	Gender	Mean age	Mechanism of rupture	Time before surgery	Type of lesion/rerupture	Associated injury/comorbidities		Type of surgery	Complications
[25] Bilateral quadriceps tendon rupture in a seasoned marathon runner with patellar spurs. (2011) Assiotis et al.	1	M	63	Tripped on a step and fell down, landing on both knees	42 days	Bilateral QT ruptures	None		Three separate Krakow-type sutures +three separate drilled tunnels in the patella and secured over the distal pole	None
[29] The hemisoleus rotational flap provides a novel superior autograft reconstructive option for the treatment of chronic extensor mechanism disruption. (2016) Auregan et al.	1	F	80	6 weeks after TKA during active extension of the knee against resistance	210 days	QT rerupture	None		Medial gastrocnemius-soleus-calcaneus rotational flap	None
[32] Allograft reconstruction of a chronic quadriceps tendon rupture with use of a novel technique. (2014) Forslund et al.	1	M	47	Primary tear: descending from a cinderblock; Re-tear: during physical therapy	1°: 210 days 2°: 365 days	Full thickness after QT rerupture	Hypertension		1°: quadriceps tendon V-Y advancement 2°: Achilles tendon-bone block allograft	None
[40] Simultaneous chronic rupture of quadriceps tendon and contra-lateral patellar tendon in a patient affected by tertiary hyperparatiroidism. (2008) Grecomoro et al.	1	M	48	Giving way of the left knee during walking and a secondary fall	40 days	Full thickness of the distal insertion of QT	Chronic renal failure, tertiary hyperparathyroidism		Codivilla’s Y/V technique	None
[41] Neglected rupture of the quadriceps tendon in a patient with chronic renal failure (case report and review of the literature). (2014) Hassani et al.	1	M	32	Common fall	60 days	Full thickness of the QT at the osteo-tendinous junction with retraction of 3 cm + calcifications	Chronic renal failure, with hemodialysis dependence for 5 years		Codivilla’s Y/V technique	None
[22] Bilateral extensor mechanism allograft reconstruction for chronic spontaneous rupture: a case report and review of the literature. (2019) Lamberti et al.	1	F	51	Acute failure of the left knee while getting up from a chair	480 days	Full-thickness lesion on the left QT	End-stage renal failure + full-thickness lesion on the right patellar tendon		A full extensor mechanism allograft	None
[36] Surgical treatment of neglected traumatic quadriceps tendon rupture with knee ankylosis. (2016) Lee et al.	1	M	15	Motorcycle accident	270 days	Chronic QT rupture + a patellar superior pole avulsion fracture of the left knee + nonunion of the left proximal tibia fracture	Open fracture of the left femur shaft + an intra-articular fracture of the proximal tibia		QT reconstruction using tibialis anterior allograft and additional screw fixation	None
[17] Reconstruction of a chronic quadriceps tendon tear in a body builder. (2006) Leopardi et al.	1	M	28	Car accident	210 days	Full-thickness tear of the quadriceps tendon proximal to the superior pole of the patella	Anabolic steroid use		QT reconstruction using gracilis and semitendinosus autograft	None
[42] Neglected ipsilateral simultaneous ruptures of patellar and quadriceps tendon (2015) Karahasanoglu et al.	1	M	40	Fall from a standing height	730 days	Chronic full-thickness and QT retraction	Ipsilateral patellar tendon rupture		Peroneus longus autograft	None
[43] Bilateral quadriceps tendon rupture and coexistent femoral neck fracture in a patient with chronic renal failure. (2007) Kazimoglu et al.	1	F	37	Two consecutive falls	60 days	Bilateral QT ruptures	Chronic renal failure, with hemodialysis for 2 years		Tycron transpatellar suture anchors	None
[28] Autologous hamstring tendon used for revision of quadiceps tendon tears. (2013) McCormick et al.	1	M	38	Primary tear: playing basketball Re-tear: fall from standing height	1°: immediatly 2°: 300 days after the rerupture	Complete QT rerupture	None		Bilateral hamstring autograft through a QT weave and a transosseous patellar repair	None
[44] Chronic Quadriceps Tendon Rupture After Total Knee Arthroplasty Augmented With Synthetic Mesh. (2017) Ormaza et al.	3	2 M 1 F	67,5	One patient experienced trauma 1 year after TKA revision surgery; one patient 6 months after TKA revision surgery; one patient 2 years after TKA revision surgery	148 days	Full thickness	One of them had a hystory of hemochromatosis		End-to-end sutures No. 5 Ethibond and reinforcement with MUTARS synthetic mesh	None
[35] Knee osteoarthritis with chronic quadriceps tendon rupture treated with total knee arthroplasty and extensor mechanism allograft reconstruction: a case report. (2018) Piatek et al.	1	M	51	Traumatic fall	90 days	Chronic full-thickness and QT retraction	Tricompartmental knee osteoarthritis		TKA + complete knee extensor mechanism allograft	None
[45] Delayed reconstruction of a quadriceps tendon. (2008) Pocock et al.	1	F	80	Common fall	2920 days	Chronic full-thickness and QT retraction	Hypertension		Four FiberWire1 (Arthrex Ltd, Sheffield, England) sutures	None
[30] Chronic rupture of the extensor apparatus of the knee joint. (2005) Poonnoose et al.	1	M	50	Common fall	4380 days	Chronic full-thickness and QT retraction	Comminuted patellar fracture treated with patellectomy		Controlateral ileo-tibial band autograft	None
[19] Quadriceps tendon repair using hamstring, prolene mesh and autologous conditioned plasma augmentation. A novel technique \for repair of chronic quadriceps tendon rupture. (2015) Rehman et al.	1	M	61	Rerupture after primary repair	300 days	Chronic full thickness	Hypertension + glaucoma		QT reconstruction using semitendinosus and gracilis autograft + prolene mesh reinforcement + PRP injection	None
[46] Chronic quadriceps rupture: treatment with lengthening and early mobilization without cerclage augmentation and a report of three cases. (2008) Rizio et al.	3	1 M 2 F	46,75	One patient fell down a flight of stairs; One patient injured his knee while jumping in church during prayers; One patient fell while stepping off a curb	240 days	Chronic full thickness	Hypertension, Hypercolesterolaemia, Obesity + obesity + hip and chronic back pain		V-Y lengthening and direct repair through drill holes in the patella without augmentation	None
[18] Repair of ruptured quadriceps tendon with Leeds-Keio ligament following revision knee surgery. (2008) Rust et al.	1	F	86	Four months after TKA revision surgery	120 days	Chronic full-thickness + 10-cm QT retraction	None		Leeds-Keio graft inserted in an 8 shape and sutured to the periosteum	None
[23] Modified V-Y turndown flap augmentation for quadriceps tendon rupture following total knee arthroplasty: a retrospective study. (2019) Shi et al.	23	10 M 13 F	61	Fall from a standing height after TKA	21 days (range, 14 to 56 days)	Complete quadriceps tendon rupture following TKA + 1rerupture	Obesity, diabetes, chronic dialysis, steroid dependence (12pt)		V-Y turndown flap	1 hematoma and delayed wound healing 1 fall and rerupture after 24 months
[47] A simultaneous bilateral quadriceps and patellar tendons rupture in patients with chronic kidney disease undergoing long-term hemodialysis: a case report. (2020) Tao et al.	2	M	33,5	1 fall down the stairs 1 sudden twist		bilateral QT ruptures	Chronic renal failure, with hemodialysis dependence for 9 and 11 years		Krackow sutures	None
[37] Extensor Mechanism Reconstruction with Use of Marlex Mesh. (2019) Abdel et al.	27	10 M 17 F	67	Rupture after TKA	219 days	Complete QT rupture	Obesity, diabetes, coronary artery diease, hypertension, OA, rheumatoid arthritis, Parkinson, cancer (leukemia, breast cancer, bladder cancer)		Marlex Mesh augmentation	5 QT re-ruptures that required mesh revision
[38] Polypropylene mesh augmentation for complete quadriceps rupture after total knee arthroplasty. (2016) Nodzo et al.	7	2 M 5 F	58,7	Rupture after TKA	90 days	Complete QT rupture	Diabetes, rheumathoid arhtritis, chronic pulmunary disease with steroid use, HCV, drug abuse, smoke, chronic renal failure		Polypropylene mesh augmentation	2 QT reruptures and 2 QT rerupture with infections
[33] Reconstruction of disrupted extensor mechanism after total knee arthroplasty. (2017) Lim et al.	3	2 M 1 F	59	Rupture after TKA	205 days	Chronic full thickness	GERD, Pulmunary embolism, diabetes, hypothyroid, asthma, hypertension, stroke, smoke		Achilles tendon allograft	1 deep infection and graft failure
[34] Long-term results of extensor mechanism reconstruction using Achilles tendon allograft after total knee arthroplasty. (2018) Wise et al.	6	3 M 3 F	68	Rupture after TKa	Complete QT rupture [5] + 1 bilateral rupture		Hypertension [3], GERD, obesity [3], hypothyroidism, asthma, chronic kidney disease, OA, diabetes [3]		Achilles tendon allograft	
[48] Quadriceps tendon rupture after total knee arthroplasty. Prevalence, complications, and outcomes. (2005) Dobbs et al.	7	1 M 6 F	72	3 patients fall, 1 patients while kneeling, 2 patients while walking, 1 patient while rising from a chair		40 days	QT rupture after TKA, 1 rerupture	Obesity [1], steroid abuse [1], DM [1]	Suture	4 reruptures and 1 chronic recurvatum

**Table 2:** Patients’ comorbidities

Nr. of reference	No. of patients	Gender	Mean age	Comorbidities
[37] Extensor mechanism reconstruction with use of Marlex Mesh. (2019) Abdel et al.	27	10 M 17 F	70	Obesity, diabetes, coronary artery diease, hypertension, OA, rheumatoid arthritis, Parkinson, cancer (leukemia, breast cancer, bladder cancer)
[25] Bilateral quadriceps tendon rupture in a seasoned marathon runner with patellar spurs. (2011) Assiotis et al.	1	M	63	None
[29] The hemisoleus rotational flap provides a novel superior autograft reconstructive option for the treatment of chronic extensor mechanism disruption. (2016) Auregan et al.	1	F	80	None
[48] Quadriceps tendon rupture after total knee arthroplasty. Prevalence, complications, and outcomes. (2005) Dobbs et al.	7	1 M 6 F	69	Obesity [1], steroid abuse [1], DM [1]
[32] Allograft reconstruction of a chronic quadriceps tendon rupture with use of a novel technique. (2014) Forslund et al.	1	M	47	Hypertension
[40] Simultaneous chronic rupture of quadriceps tendon and contra-lateral patellar tendon in a patient affected by tertiary hyperparatiroidism. (2008) Grecomoro et al.	1	M	48	Chronic renal failure, tertiary hyperparathyroidism
[41] Neglected rupture of the quadriceps tendon in a patient with chronic renal failure (case report and review of the literature). (2014) Hassani et al.	1	M	32	CKD with hemodialysis dependence for 5 years
[22] Bilateral extensor mechanism allograft reconstruction for chronic spontaneous rupture: a case report and review of the literature. (2019) Lamberti et al.	1	F	51	End-stage CKD
[36] Surgical treatment of neglected traumatic quadriceps tendon rupture with knee ankylosis. (2016) Lee et al.	1	M	15	Open fracture of the left femur shaft, intra-articular fracture of the proximal tibia
[17] Reconstruction of a chronic quadriceps tendon tear in a body builder. (2006) Leopardi et al.	1	M	28	Anabolic steroid use
[33] Reconstruction of disrupted extensor mechanism after total knee arthroplasty. (2017) Lim et al.	3	2 M 1 F	69	GERD, pulmonary embolism, diabetes, hypothyroidism, asthma, hypertension, stroke, smoke
[42] Neglected ipsilateral simultaneous ruptures of patellar and quadriceps tendon (2015) Karahasanoglu et al.	1	M	40	Ipsilateral patellar tendon rupture
[43] Bilateral quadriceps tendon rupture and coexistent femoral neck fracture in a patient with chronic renal failure. (2007) Kazimoglu et al.	1	F	37	CKD, with hemodialysis dependence for 2 years
[28] Autologous hamstring tendon used for revision of quadiceps tendon tears. (2013) McCormick et al.	1	Male	38	None
[38] Polypropylene mesh augmentation for complete quadriceps rupture after total knee arthroplasty. (2016) Nodzo et al.	7	2 M 5 F	56	Diabetes, rheumathoid arhtritis, chronic pulmonary disease with steroid use, HCV, drug abuse, smoke, chronic renal failure
[44] Chronic quadriceps tendon rupture after total knee arthroplasty augmented with synthetic mesh. (2017) Ormaza et al.	3	2 M 1 F	70	Hemochromatosis
[35] Knee osteoarthritis with chronic quadriceps tendon rupture treated with total knee arthroplasty and extensor mechanism allograft reconstruction: a case report. (2018) Piatek et al.	1	M	51	Tricompartmental knee osteoarthritis
[45] Delayed reconstruction of a quadriceps tendon. (2008) Pocock et al.	1	F	80	Hypertension
[30] Chronic rupture of the extensor apparatus of the knee joint. (2005) Poonnoose et al.	1	M	50	Comminuted patellar fracture treated with patellectomy
[19] Quadriceps tendon repair using hamstring, prolene mesh and autologous conditioned plasma augmentation. A novel technique for repair of chronic quadriceps tendon rupture. (2015) Rehman et al.	1	M	61	Hypertension, glaucoma
[46] Chronic quadriceps rupture: treatment with lengthening and early mobilization without cerclage augmentation and a report of three cases. (2008) Rizio et al.	3	1 M 2 F	50	Hypertension, Hypercolesterolaemia, Obesity, and chronic back pain
[18] Repair of ruptured quadriceps tendon with Leeds-Keio ligament following revision knee surgery. (2008) Rust et al.	1	F	86	None
[23] Modified V-Y turndown flap augmentation for quadriceps tendon rupture following total knee arthroplasty: a retrospective study. (2019) Shi et al.	23	10 M 13F	61	Obesity, diabetes, chronic dialysis, steroid dependence (12pt)
[47] A simultaneous bilateral quadriceps and patellar tendons rupture in patients with chronic kidney disease undergoing long-term hemodialysis: a case report. (2020) Tao et al.	2	M	33,5	Chronic renal failure, with hemodialysis dependence for 9 and 11 years
[34] Long-term results of extensor mechanism reconstruction using Achilles tendon allograft after total knee arthroplasty. (2018) Wise et al.	6	3 M 3 F	69	Hypertension [3], GERD, obesity [3], hypothyroidism, asthma, chronic kidney disease, OA, diabetes [3]

**Table 3:** QTR in patient previously treated with TKA

Nr. of reference	No. of patients	Gender	Mean age	Mechanism of rupture	Time before surgery	Type of lesion/rerupture	Associated injury/comorbidities	Type of surgery	Complications
[44] Chronic quadriceps tendon rupture after total knee arthroplasty augmented with synthetic mesh. (2017) Ormaza et al.	3	2 M 1 F	67,5	One of them experienced trauma 1 year after TKA revision surgery; one of them 6 months after TKA revision surgery; one of them 2 years after TKA revision surgery	148 days	Full thickness	One of them had a hystory of hemochromatosis	End-to-end sutures No. 5 Ethibond and reinforcement with MUTARS synthetic mesh	None
[18] Repair of ruptured quadriceps tendon with Leeds-Keio ligament following revision knee surgery. (2008) Rust et al.	1	F	86	4 months after TKA revision surgery	120 days	Chronic full-thickness + 10-cm QT retraction	None	Leeds-Keio graft inserted in an 8 shape and sutured to the periosteum	None
[23] Modified V-Y turndown flap augmentation for quadriceps tendon rupture following total knee arthroplasty: a retrospective study. (2019) Shi et al.	23	10 M 13F	61	Fall from a standing height after TKA	21 days (range, 14 to 56 days)	Complete quadriceps tendon rupture following TKA + 1rerupture	Obesity, diabetes, chronic dialysis, steroid dependence (12pt)	V-Y turndown flap	1 hematoma and delayed wound healing 1 fall and rerupture after 24 months
[37] Extensor mechanism reconstruction with use of Marlex Mesh. (2019) Abdel et al.	27	10 M 17 F	67	Rupture after TKA	219	Complete QT rupture	Obesity, diabetes, coronary artery diease, hypertension, OA, rheumatoid arthritis, Parkinson, cancer (leukemia, breast cancer, bladder cancer)	Marlex Mesh augmentation	5 QT re-ruptures that required mesh revision
[38] Polypropylene mesh augmentation for complete quadriceps rupture after total knee arthroplasty. (2016) Nodzo et al.	7	2 M 5 F	58,7	Rupture after TKA	90 days	Complete QT rupture	Diabetes, rheumathoid arhtritis, chronic pulmunary disease with steroid use, HCV, drug abuse, smoke, chronic renal failure	Polypropylene mesh augmentation	2 QT reruptures and 2 QT rerupture with infections
[33] Reconstruction of disrupted extensor mechanism after total knee arthroplasty. (2017) Lim et al.	3	2 M 1 F	59	Rupture after TKA	205 days	Complete QT rupture	GERD, Pulmunary embolism, diabetes, hypothyroid, asthma, hypertension, stroke, smoke	Achilles tendon allograft	1 deep infection and graft failure
[34] Long-term results of extensor mechanism reconstruction using Achilles tendon allograft after total knee arthroplasty. (2018) Wise et al.	6	3 M 3 F	68	Rupture after TKa	Complete QT rupture [5] + 1 bilateral rupture		Hypertension [3],GERD, obesity [3], hypothyroidism, asthma, chronic kidney disease, OA, Diabetes [3]	Achilles tendon allograft	None
[48] Quadriceps tendon rupture after total knee arthroplasty. Prevalence, complications, and outcomes. (2005) Dobbs et al.	7	1 M 6 F	72	3 patients fall, 1 patients while kneeling, 2 patients while walking, 1 patient while rising from a chair	40 days	QT rupture after TKA, 1 rerupture	Obesity [1], steroid abuse [1], DM [1]	Suture	4 reruptures and 1 chronic recurvatum

## Discussion

According to the main findings of the present systematic review, mostly chronic ruptures after TKA are reported in the literature (72 patients, 74.5%), followed by chronic QTRs (16 patients, 16.5%) and re-ruptures (9 patients, 9%). Synthetic augmentation was the most frequently used technique in chronic QTRs after primary TKA, while the use of tendon grafts was preferred in chronic QTRs and re-ruptures. After surgical treatment, the most commonly reported complications were re-ruptures (9 patients, 9%), deep infections (3 patients, 3%), and one patient (1%) developed knee recurvatum. Twenty-one patients (22%) reported an extensor lag, while 48 (50%) showed a decreased active flexion at the latest follow-up. The quadriceps tendon, given its structural and biomechanics properties, can sustain high loads without rupture. However, severe degenerative changes can impact the tendon and can be age related or caused by systemic conditions [24, 25]. It should not be surprising, therefore, that 73% of the patients from the articles included in the current study suffered from cardiovascular and metabolic conditions, such as hypertension, diabetes, obesity, and chronic renal failure. Furthermore, previous surgery such as TKA can represent a risk factor, probably because of previous insult to the tendon structure [26]. Surgical treatment of chronic QTRs can be challenging because of the large defect and/or tissue degeneration in the substance of the tendon (Table 4). While ruptures of other tendons (Table 5), such as the flexor digitorum profundus and superficialis tendons, require swift intervention because of the prompt retraction of the tendon stumps, in extensor tendons, such as the triceps brachii and quadriceps tendon, retraction is slower, and usually can be treated acutely with direct repair [27]. Recently, a systematic review about chronic QTRs suggested that the timing of surgical intervention plays a crucial role in the functional outcomes, setting the cutoff for early treatment at 2–3 weeks from the injury [15]. However, all the articles included in the current study reported acceptable functional outcomes even though the surgery had occurred at least 40 days after the rupture. In this systematic review, only 16% of the patients were treated with direct suture. Augmentation techniques were employed in 82 patients (84%) using synthetic mesh (39%), autografts (12.5%), allografts (13.5%), and V-Y lengthening (29%) (Table 6). The autologous hamstring and peroneus longus (PL) tendon grafts were the most commonly used autografts in chronically retracted and re-ruptured tendons: McCormick and Rehman both performed a bilateral hamstring autograft to restore a large substance defect caused by a re-rupture of the quadriceps tendon after primary repair, and Rehman et al. used reinforcement with a prolene mesh and PRP injection [19, 28]. However, comparing results in the studies reporting the outcomes of surgery where both gracilis and semitendinosus had been used, McCormick et al. reported better results in terms of clinical outcomes (ROM, active flexion and lag absence) and return to daily activities, while Leopardi et al. and Rehman et al. reported an average 7.5° extensor lag at follow-up [17, 19, 28]. Other autologous grafts have been used, such as the contralateral ilio-tibial (IT) band and a medial gastrocnemius-soleus rotational flap. All of these produced good functional results with no further complication, except for one patient treated with contralateral IT band graft who reported a 40° extensor lag at 9 months follow-up [29, 30]. Tendon augmentation with an allograft was used in 13 patients (13%), mostly in QTRs after prior TKA, and Achilles tendon (AT) bone block allografts were most frequently used (ten patients, 11%): Wise et al. and Lim et al. treated six and three patients respectively with an Achilles tendon allograft, showing better results compared to the full extensor mechanism allograft reported by Burnett et al. [31] Even though the Achilles tendon is often considered the most suitable allograft option, the patients included in the current study treated with AT allografts reported an average 12° extensor lag at follow-up, with graft failure from deep infection in one case (1%) [32–34]. Complete extensor-mechanism allografting was performed in two patients (2%), with no extension lag, full active extension, and no further complications except for a skin ulcer in one case [22, 35]. One patient (1%) was treated with a tibialis anterior allograft, showing good functional results at the latest follow-up [36]. Furthermore, 35 patients (36%) with a complete QTR after TKA underwent synthetic mesh tendon reconstruction, but reported nine re-ruptures, of which five required a MESH revision and 2 developed a deep infection [37, 38]. The past medical history plays a crucial role in the choice of surgical strategy: in patients with chronic QTRs with no history of TKA, end-to-end sutures produce a good clinical outcome, even if the timing of the rupture is longer than 6 weeks. On the other hand, in patients who have undergone a TKA, direct repairs produce unpredictable outcomes, and the repair often needs to be reinforced [31]. The great discrepancy in results following treatment of patients with or without a TKA can be explained by the older age and comorbidities of the TKA group: in the current study, the mean age of patients with chronic QTRs was 44, while the mean age of the patients with chronic QTRs following TKA was 68.5 years old. Furthermore, a part of this discrepancy may be explained by the impaired quadriceps blood supply, reduced by the arthrotomy used to expose the joint [39]. Nine patients (9%) treated with mesh augmentation endured a re-rupture which required revision in five cases, while deep infection and re-rupture occurred in two of them (2%) with graft failure. One patient, treated with an Achilles tendon allograft, reported graft failure as a consequence of deep infection. Furthermore, four patients (4%) treated with late suture repair for QTR after TKA experienced a re-rupture. Further prospective studies are needed to establish a clear rehabilitation protocol for chronic QTRs, QTRs after TKA, and re-ruptures. Usually, published studies evaluate surgical outcomes through scales and scoring systems such as VAS, KSS, IKDC, and Lysholm Score, and quadriceps strength is measured with isokinetic tests on both the healthy and the treated legs. In the current study, only eight of 25 studies reported the use of a scoring system: in six studies, the Knee Society Score was assessed, while in two studies the Lysholm score was used. Furthermore, from the analysis of the reported follow-up, it emerged that only 12 studies evaluated the return to daily activities, while only Leopardi et al. [17] and McCormick et al. [28] documented the return to sport. While several protocols are available for acute QTR rehabilitation, there is a lack of studies about rehabilitation and follow-up after treatment of chronic ruptures, ruptures after TKA, and re-ruptures. In this study, the mean period of immobilization reported was 5.7 weeks, even though Abdel et al. and Nodzo et al., who performed tendon augmentation using synthetic mesh in patients with QTR after TKA, reported longer immobilization periods of respectively 12 and 8 weeks [37, 38]. The choice of the best procedure is closely linked to the type of lesion. The size of the substance loss and the quality of the tendon stumps appear to be the most relevant factors in the choice of surgical management of these ruptures, rather than the timing of the rupture. Chronic QTRs and re-ruptures were usually treated with grafts. Autografts showed better functional outcomes and lower complications rates than allografts. Ruptures following TKA were mainly treated with synthetic augmentation techniques, with the highest rate of re-ruptures and complications. Although autografts appear to be the most suitable option to treat complex QTRs, further comparative studies are needed to guide the choice of the graft and the best surgical technique.

**Table 4:** Chronic QTR

Nr. of reference	No. of patients	Gender	Mean age	Mechanism of rupture	Time before surgery	Type of lesion/rerupture	Associated injury/comorbidities	Type of surgery
[25] Bilateral quadriceps tendon rupture in a seasoned marathon runner with patellar spurs. (2011) Assiotis et al.	1	M	63	Tripped on a step and fell down, landing on both his knees	42 days	Bilateral QT ruptures	None	Three separate Krakow-type sutures +three separate drilled tunnels in the patella and secured over the distal pole
[40] Simultaneous chronic rupture of quadriceps tendon and contra-lateral patellar tendon in a patient affected by tertiary hyperparatiroidism. (2008) Grecomoro et al.	1	M	48	Subsiding of the left knee during walking and a secondary fall	40 days	Full thickness of the distal insertion of QT	Chronic renal failure, tertiary hyperparathyroidism	Codivilla’s Y/V technique
[41] Neglected rupture of the quadriceps tendon in a patient with chronic renal failure (case report and review of the literature). (2014) Hassani et al.	1	M	32	Common fall	60 days	Full thickness of the QT at the osteo-tendinous junction with retraction of 3 cm + calcifications	Chronic renal failure, with hemodialysis dependence for 5 years	Codivilla’s Y/V technique
[22] Bilateral extensor mechanism allograft reconstruction for chronic spontaneous rupture: a case report and review of the literature. (2019) Lamberti et al.	1	F	51	Acute failureof the left knee while getting up from a chair	480 days	Full-thickness lesion on the left QT	End-stage renal failure + full-thickness lesion on the right patellar tendon	A full extensor mechanism allograft
[36] Surgical treatment of neglected traumatic quadriceps tendon rupture with knee ankylosis. (2016) Lee et al.	1	M	15	Motorcycle traffic accident	270 days	Chronic QT rupture + a patellar superior pole avulsion fracture of the left knee + nonunion of the left proximal tibia fracture	Open fracture of the left femur shaft + an intra-articular fracture of the proximal tibia	QT reconstruction using tibialis anterior allograft and additional screw fixation
[17] Reconstruction of a chronic quadriceps tendon tear in a body builder. (2006) Leopardi et al.	1	M	28	Car accident	210 days	Full-thickness tear of the quadriceps tendon proximal to the superior pole of the patella	Anabolic steroid use	QT reconstruction using gracilis and semitendinosus autograft
[42] Neglected ipsilateral simultaneous ruptures of patellar and quadriceps tendon (2015) Karahasanoglu et al.	1	M	40	Fall from a standing height	730 days	Chronic full-thickness and QT retraction	Ipsilateral patellar tendon rupture	Peroneus longus autograft
[43] Bilateral quadriceps tendon rupture and coexistent femoral neck fracture in a patient with chronic renal failure. (2007) Kazimoglu et al.	1	F	37	Two consecutive falls	60 days	bilateral QT ruptures	Chronic renal failure, with hemodialysis dependence for 2 years	Tycron transpatellar suture anchors
[35] Knee osteoarthritis with chronic quadriceps tendon rupture treated with total knee arthroplasty and extensor mechanism allograft reconstruction: a case report. (2018) Piatek et al.	1	M	51	Traumatic fall	90 days	Chronic full-thickness and QT retraction	Tricompartmental knee osteoarthritis	TKA + complete knee extensor mechanism allograft
[45] Delayed reconstruction of a quadriceps tendon. (2008) Pocock et al.	1	F	80	Common fall	2920 days	Chronic full-thickness and QT retraction	Hypertension	Four FiberWire1 (Arthrex Ltd, Sheffield, England) sutures
[30] Chronic rupture of the extensor apparatus of the knee joint. (2005) Poonnoose et al.	1	M	50	Common fall	4380 days	Chronic full-thickness and QT retraction	Comminuted patellar fracture treated with patellectomy	Controlateral ileo-tibial band autograft
[46] Chronic quadriceps rupture: treatment with lengthening and early mobilization without cerclage augmentation and a report of three cases. (2008) Rizio et al.	3	1 M 2 F	46,75	One of them down a flight of stairs; One of them injured his knee while jumping in church during prayers; One of them fell while stepping off a curb.	240 days	Chronic full thickness	Hypertension, hypercolesterolaemia, obesity + obesity + hyp and chronic back pain	V-Y lengthening and direct repair through drill holes in the patella without augmentation
[47] A simultaneous bilateral quadriceps and patellar tendons rupture in patients with chronic kidney disease undergoing long-term hemodialysis: a case report. (2020) Tao et al.	2	M	33,5	1 fall down the stairs 1 sudden twist		Bilateral QT ruptures	Chronic renal failure, with hemodialysis dependence for 9 and 11 years	Krackow sutures

**Table 5:** Re-rupture of quadriceps tendon

Nr. of reference	No. of patients	Gender	Mean age	Mechanism of rupture	Time before surgery	Type of lesion/rerupture	Associated injury/comorbidities	Type of surgery
[29] The hemisoleus rotational flap provides a novel superior autograft reconstructive option for the treatment of chronic extensor mechanism disruption. (2016) Auregan et al.	1	F	80	6 weeks after TKA during active extension of the knee against resistance	210 days	QT rerupture	None	Medial gastrocnemius-soleus-calcaneus rotational flap
[32] Allograft reconstruction of a chronic quadriceps tendon rupture with use of a novel technique. (2014) Forslund et al.	1	M	47	1°: descending from a cinderblock 2°: during physical therapy	1°: 210 days 2°: 365 days	Full thickness after QT rerupture	Hypertension	1°: quadriceps tendon V-Y advancement 2°: Achilles tendon-bone block allograft
[28] Autologous hamstring tendon used for revision of quadiceps tendon tears. (2013) McCormick et al.	1	M	38	1°: playing basketball 2°: a fall from standing height	1°: immediatly 2°: 300 days after the rerupture	Complete QT rerupture	None	Bilateral hamstring autograft through a QT weave and a transosseous patellar repair
[23] Modified V-Y turndown flap augmentation for quadriceps tendon rupture following total knee arthroplasty: a retrospective study. (2019) Shi et al.	1		61	Fall from a standing height after TKA	720 days	Complete QT rerupture	None	V-Y turndown flap
[19] Quadriceps tendon repair using hamstring, prolene mesh and autologous conditioned plasma augmentation. A novel technique for repair of chronic quadriceps tendon rupture. (2015) Rehman et al.	1	M	61	Rerupture after primary repair	300 days	Complete QT rerupture	Hypertension and glaucoma	Semitendinosus and gracilis autograft + prolene mesh reinforcement + PRP injection
[48] Quadriceps tendon rupture after total knee arthroplasty. Prevalence, complications, and outcomes. (2005) Dobbs et al.	4	1 M 3 F	69 M 60 F	3 fall, 1 rising from a chair	40 days	Complete QT rerupture	1 DM, 1 steroids, 2 previous knee surgeries	Sutures

**Table 6:** Surgical technique used in QTR treatment

Type of surgery	Nr. of reference	No. of patients	Gender	Mean age	Mean range time before surgery	
Codivilla’s Y/V technique						
	[40] Simultaneous chronic rupture of quadriceps tendon and contra-lateral patellar tendon in a patient affected by tertiary hyperparatiroidism. (2008) Grecomoro et al.	1	M	48	40 days	Fig. 2 and Fig. 3
	[41] Neglected rupture of the quadriceps tendon in a patient with chronic renal failure (case report and review of the literature). (2014) Hassani et al.	1	M	32	60 days	
	[46] Chronic quadriceps rupture: treatment with lengthening and early mobilization without cerclage augmentation and a report of three cases. (2008) Rizio et al.	3	1 M 2 F	46,75	240 days	
	[23] Modified V-Y turndown flap augmentation for quadriceps tendon rupture following total knee arthroplasty: a retrospective study. (2019) Shi et al.	23	10 M 13 F	61	21 days	
Autograft						
Medial gastrocnemius-soleus-calcaneus rotational flap	[29] The hemisoleus rotational flap provides a novel superior autograft reconstructive option for the treatment of chronic extensor mechanism disruption. (2016) Auregan et al.	1	F	80	210 days	Fig. 4
Peroneus longus	[42] Neglected ipsilateral simultaneous ruptures of patellar and quadriceps tendon (2015) Karahasanoglu et al.	1	M	40	730 days	Fig. 5
Gracilis and semitendinosus	[17] Reconstruction of a chronic quadriceps tendon tear in a body builder. (2006) Leopardi et al.	1	M	28	210 days	
Gracilis and semitendinosus with prolene mesh reinforcement	[28] Autologous hamstring tendon used for revision of quadiceps tendon tears. (2013) McCormick et al.	1	M	38	1°: immediatly 2°: 300 days after the rerupture	Fig. 6
	[19] Quadriceps tendon repair using hamstring, prolene mesh and autologous conditioned plasma augmentation. A novel technique for repair of chronic quadriceps tendon rupture. (2015) Rehman et al.	1	M	61	300 days	
Ileo-tibial band	[30] Chronic rupture of the extensor apparatus of the knee joint. (2005) Poonnoose et al.	1	M	50	4380 days	
Allograft						
Achilles tendon-bone block	[32] Allograft reconstruction of a chronic quadriceps tendon rupture with use of a novel technique. (2014) Forslund et al.	1	M	47	365 days	
	[33] Reconstruction of disrupted extensor mechanism after total knee arthroplasty. (2017) Lim et al.	3	2 M 1 F	59	205 days	
	[34] Long-term results of extensor mechanism reconstruction using Achilles tendon allograft after total knee arthroplasty. (2018) Wise et al.	6	3 M 3 F	68		
Full extensor mechanism	[22] Bilateral extensor mechanism allograft reconstruction for chronic spontaneous rupture: a case report and review of the literature. (2019) Lamberti et al.	1	F	51	480 days	
	[35] Knee osteoarthritis with chronic quadriceps tendon rupture treated with total knee arthroplasty and extensor mechanism allograft reconstruction: a case report. (2018) Piatek et al.	1	M	51	90 days	Fig. 7
Tibialis anterior	[36] Surgical treatment of neglected traumatic quadriceps tendon rupture with knee ankylosis. (2016) Lee et al.	1	M	15	270 days	
Synthetic mesh						
	[37] Extensor mechanism reconstruction with use of Marlex Mesh. (2019) Abdel et al.	27	10 M 17 F	67	219 days	Fig. 8
	[38] Polypropylene mesh augmentation for complete quadriceps rupture after total knee arthroplasty. (2016) Nodzo et al.	7	2 M 5 F	58,7	90 days	
	[18] Repair of ruptured quadriceps tendon with Leeds-Keio ligament following revision knee surgery. (2008) Rust et al.	1	F	86	120 days	
Sutures						
	[25] Bilateral quadriceps tendon rupture in a seasoned marathon runner with patellar spurs. (2011) Assiotis et al.	1	M	63	42 days	
	[43] Bilateral quadriceps tendon rupture and coexistent femoral neck fracture in a patient with chronic renal failure. (2007) Kazimoglu et al.	1	F	37	60 days	
	[44] Chronic quadriceps tendon rupture after total knee arthroplasty augmented with synthetic mesh. (2017) Ormaza et al.	3	2 M 1 F	67,5	148 days	
	[45] Delayed reconstruction of a quadriceps tendon. (2008) Pocock et al.	1	F	80	2920 days	
	[47] A simultaneous bilateral quadriceps and patellar tendons rupture in patients with chronic kidney disease undergoing long-term hemodialysis: a case report. (2020) Tao et al.	2	M	33,5		
	[48] Quadriceps tendon rupture after total knee arthroplasty. Prevalence, complications, and outcomes. (2005) Dobbs et al.	7	1 M 6 F	72	40 days	

This study has several limitations. First, the retrospective design and the lack of blinding of most of the included studies. The included studies did not mention the type of prosthesis. For this reason, it was impossible to compare the outcomes of QT repair in unicompartimental or total knee arthroplasty, and reported different surgical approaches without a general consensus on the best surgical management, which could represent an important source of bias. Given these limitations, results from the present study must therefore be interpreted with caution. Future studies should compare surgical procedures to establish a gold standard for the treatment of complex QTRs.

## Conclusions

Complex ruptures of the QT can be chronic ruptures, re-ruptures, or ruptures that occurred after TKA. Augmentation techniques are usually chosen to deal with loss or poor quality of tendon substance. Autografts have shown good functional outcomes in the treatment of complex QTRs. However, given the lack of evidence-based recommendations, surgeons’ experience seems to be the key factor in the choice of the most appropriate procedure.

## Supplementary Information

**Additional file 1.** Prisma checklist.

## Data Availability

This study does not contain any third material.

## Abbreviations

TKA: Total knee arthroplasty
QT: Quadriceps tendon
QTR: Quadriceps tendon rupture
PRISMA: Preferred Reporting Items for Systematic Reviews and Meta-Analyses

## References

[1] Ilan DI, Tejwani N, Keschner M, Leibman M (2003). Quadriceps tendon rupture. J Am Acad Orthop Surg.

[2] Clayton RA, Court-Brown CM (2008). The epidemiology of musculoskeletal tendinous and ligamentous injuries. Injury.

[3] Scuderi C (1958). Ruptures of the quadriceps tendon; study of twenty tendon ruptures. Am J Surg.

[4] Shah MK (2002). Simultaneous bilateral rupture of quadriceps tendons: analysis of risk factors and associations. South Med J.

[5] Khaliq Y, Zhanel GG (2005). Musculoskeletal injury associated with fluoroquinolone antibiotics. Clin Plast Surg.

[6] Aracil J, Salom M, Aroca JE, Torro V, Lopez-Quiles D (1999). Extensor apparatus reconstruction with Leeds-Keio ligament in total knee arthroplasty. J Arthroplasty.

[7] Lynch AF, Rorabeck CH, Bourne RB (1987). Extensor mechanism complications following total knee arthroplasty. J Arthroplasty.

[8] Maffulli N, Spiezia F, La Verde L (2017). The management of extensor mechanism disruption after total knee arthroplasty: a systematic review. Sports Med Arthrosc Rev.

[9] Yepes H, Tang M, Morris SF, Stanish WD (2008). Relationship between hypovascular zones and patterns of ruptures of the quadriceps tendon. J Bone Joint Surg Am.

[10] Siwek CW, Rao JP (1981). Ruptures of the extensor mechanism of the knee joint. J Bone Joint Surg Am.

[11] Spector ED, DiMarcangelo MT, Jacoby JH (1995). The radiologic diagnosis of quadriceps tendon rupture. N J Med.

[12] McLaughlin HL, Francis KC (1956). Operative repair of injuries to the quadriceps extensor mechanism. Am J Surg.

[13] Brossard P, Le Roux G, Vasse B (2017). Acute quadriceps tendon rupture repaired by suture anchors: Outcomes at 7 years' follow-up in 25 cases. Orthop Traumatol Surg Res.

[14] Hochheim M, Bartels E, Iversen J (2019). Quadriceps tendon rupture. Anchor or transosseous suture? A systematic review. Muscle Ligaments and Tendons. Journal.

[15] Elattar O, McBeth Z, Curry EJ, Parisien RL, Galvin JW, Li X. Management of chronic quadriceps tendon rupture: a critical analysis review. JBJS Rev. 2021;9(5). 10.2106/JBJS.RVW.20.00096. 10.2106/JBJS.RVW.20.00096 33956669

[16] Katzman BM, Silberberg S, Caligiuri DA, Klein DM, DiPaolo P (1997). Delayed repair of a quadriceps tendon. Orthopedics.

[17] Leopardi P, Vico G, Rosa D (2006). Reconstruction of a chronic quadriceps tendon tear in a body builder. Knee Surg Sports Traumatol Arthrosc.

[18] Rust PA, Tanna N, Spicer DD (2008). Repair of ruptured quadriceps tendon with Leeds-Keio ligament following revision knee surgery. Knee Surg Sports Traumatol Arthrosc.

[19] Rehman H, Kovacs P (2015). Quadriceps tendon repair using hamstring, prolene mesh and autologous conditioned plasma augmentation A novel technique for repair of chronic quadriceps tendon rupture. Knee.

[20] Moher D, Liberati A, Tetzlaff J, Altman DG, for the PRISMA Group (2009). Preferred reporting items for systematic reviews and meta-analyses: the PRISMA statement. BMJ.

[21] Howick JCI, Glasziou P, Greenhalgh T, Heneghan C, Liberati A, Moschetti I, Phillips B, Thornton H, Goddard O, Hodgkinson M (2011). The 2011 Oxford CEBM Levels of Evidence. Oxford Centre for Evidence-Based Medicine.

[22] Lamberti A, Loconte F, Spinarelli A, Baldini A (2019). Bilateral extensor mechanism allograft reconstruction for chronic spontaneous rupture: a case report and review of the literature. JBJS Case Connect.

[23] Shi SM, Shi GG, Laurent EM, Ninomiya JT (2019). Modified V-Y turndown flap augmentation for quadriceps tendon rupture following total knee arthroplasty: a retrospective study. J Bone Joint Surg Am.

[24] Garner MR, Gausden E, Berkes MB, Nguyen JT, Lorich DG (2015). Extensor mechanism injuries of the knee: demographic characteristics and comorbidities from a review of 726 patient records. J Bone Joint Surg Am.

[25] Assiotis A, Pengas I, Vemulapalli K. Bilateral quadriceps tendon rupture in a seasoned marathon runner with patellar spurs. Grand Rounds. 2011:11.

[26] Ng J, Balcells-Nolla P, James PJ, Bloch BV (2021). Extensor mechanism failure in total knee arthroplasty. EFORT Open Rev.

[27] Oliva F, Sesti FF, Gasparini M (2020). Chronic distal triceps brachii tendon ruptures. A systematic review of surgical procedures and outcomes. Muscle Ligaments and Tendons Journal.

[28] McCormick F, Nwachukwu BU, Kim J, Martin SD (2013). Autologous hamstring tendon used for revision of quadiceps tendon tears. Orthopedics.

[29] Auregan JC, Lin JD, Lombardi JM, Jang E, Macaulay W, Rosenwasser MP (2016). The hemisoleus rotational flap provides a novel superior autograft reconstructive option for the treatment of chronic extensor mechanism disruption. Arthroplast Today.

[30] Poonnoose PM, Korula RJ, Oommen AT (2005). Chronic rupture of the extensor apparatus of the knee joint. Med J Malaysia.

[31] Burnett RS, Berger RA, Paprosky WG (2004). Extensor mechanism allograft reconstruction after total knee arthroplasty A comparison of two techniques. J Bone Joint Surg Am.

[32] Forslund J, Gold S, Gelber J (2014). Allograft reconstruction of a chronic quadriceps tendon rupture with use of a novel technique. JBJS Case Connect.

[33] Lim CT, Amanatullah DF, Huddleston JI (2017). Reconstruction of disrupted extensor mechanism after total knee arthroplasty. J Arthroplasty.

[34] Wise BT, Erens G, Pour AE, Bradbury TL, Roberson JR (2018). Long-term results of extensor mechanism reconstruction using Achilles tendon allograft after total knee arthroplasty. Int Orthop.

[35] Piatek AZ, Lee P, DeRogatis MJ (2018). Knee osteoarthritis with chronic quadriceps tendon rupture treated with total knee arthroplasty and extensor mechanism allograft reconstruction: a case report. JBJS Case Connect.

[36] Lee SH, Song EK, Seon JK, Woo SH (2016). Surgical treatment of neglected traumatic quadriceps tendon rupture with knee ankylosis. Knee Surg Relat Res.

[37] Abdel MP, Pagnano MW, Perry KI, Hanssen AD (2019). Extensor mechanism reconstruction with use of Marlex mesh. JBJS Essent Surg Tech.

[38] Nodzo SR, Rachala SR (2016). Polypropylene mesh augmentation for complete quadriceps rupture after total knee arthroplasty. Knee.

[39] Ritter MA, Herbst SA, Keating EM, Faris PM, Meding JB (1996). Patellofemoral complications following total knee arthroplasty Effect of a lateral release and sacrifice of the superior lateral geniculate artery. J Arthroplasty.

[40] Grecomoro G, Camarda L, Martorana U (2008). Simultaneous chronic rupture of quadriceps tendon and contra-lateral patellar tendon in a patient affected by tertiary hyperparatiroidism. J Orthop Traumatol.

[41] Hassani ZA, Boufettal M, Mahfoud M (2014). Neglected rupture of the quadriceps tendon in a patient with chronic renal failure (case report and review of the literature). Pan Afr Med J.

[42] Karahasanoglu I, Yologlu O, Kerimoglu S (2015). Neglected ipsilateral simultaneous ruptures of patellar and quadriceps tendon. Eklem Hastalik Cerrahisi.

[43] Kazimoglu C, Yagdi S, Karapinar H (2007). Bilateral quadriceps tendon rupture and coexistent femoral neck fracture in a patient with chronic renal failure. Acta Orthop Traumatol Turc.

[44] Ormaza A, Moreta J, Mosquera J, de Ugarte OS, JLMD M (2017). Chronic quadriceps tendon rupture after total knee arthroplasty augmented with synthetic mesh. Orthopedics.

[45] Pocock CA, Trikha SP, Bell JS (2008). Delayed reconstruction of a quadriceps tendon. Clin Orthop Relat Res.

[46] Rizio L, Jarmon N (2008). Chronic quadriceps rupture: treatment with lengthening and early mobilization without cerclage augmentation and a report of three cases. J Knee Surg.

[47] Tao Z, Liu W, Ma W, Luo P, Zhi S, Zhou R (2020). A simultaneous bilateral quadriceps and patellar tendons rupture in patients with chronic kidney disease undergoing long-term hemodialysis: a case report. BMC Musculoskelet Disord.

[48] Dobbs RE, Hanssen AD, Lewallen DG, Pagnano MW (2005). Quadriceps tendon rupture after total knee arthroplasty. Prevalence, complications, and outcomes. J Bone Joint Surg Am.

